# Flunixin Meglumine Enhanced Bone Fracture Healing in Rabbits Associated with Activation of Early Collagen Deposition and Enhancement of Vascular Endothelial Growth Factor Expression

**DOI:** 10.3390/ani11102834

**Published:** 2021-09-28

**Authors:** Mohamed Elgendy, Gamal Elsayad, Magdi Seleim, Walied Abdo, Roua S. Baty, Ehab Kotb Elmahallawy, Ayman Atiba

**Affiliations:** 1 Department of Surgery, Anesthesiology and Radiology, Faculty of Veterinary Medicine, Kafrelsheikh University, Kafrelsheikh 33516, Egypt; Mohamed_elgendy@vet.kfs.edu.eg (M.E.); elsayad@vet.kfs.edu.eg (G.E.); magdy.saleem@vet.kfs.edu.eg (M.S.); 2 Department of Pathology, Faculty of Veterinary Medicine, Kafrelsheikh University, Kafr Elsheikh 33516, Egypt; walid.eid@vet.kfs.edu.eg; 3 Department of Biotechnology, College of Science, Taif University, P.O. Box 11099, Taif 21944, Saudi Arabia; rsbaty@tu.edu.sa; 4 Department of Zoonoses, Faculty of Veterinary Medicine, Sohag University, Sohag 82524, Egypt

**Keywords:** flunixin meglumine, ketoprofen, bone healing, fracture, rabbit

## Abstract

**Simple Summary:**

Postfracture treatment with nonsteroidal anti-inflammatory drugs might result in delayed healing. This study was aimed to evaluate and compare the effects of flunixin meglumine (FM) and ketoprofen on bone fracture healing in rabbits. A simple unilateral diaphyseal fracture was made and followed by fixation by K-wire. Healing was evaluated with radiography, histopathology, and immunohistochemistry. Interestingly, the results revealed that FM enhanced bone fracture healing combined with the activation of early collagen deposition, marked angiogenesis, and enhanced vascular endothelial growth factor. However, ketoprofen delayed bone fracture healing. These findings provide novel baseline information about the potential beneficial effects of FM on bone fracture healing cases.

**Abstract:**

Nonsteroidal anti-inflammatory drugs (NSAIDs) are among the most commonly used postoperative analgesics, antipyretics, and anti-inflammatories, and they help prevent blood clotting. However, most NSAIDs delay bone healing. This study was aimed to investigate bone healing in a rabbit animal model by assessing the ability of flunixin meglumine (FM) and ketoprofen to induce fracture healing by examining histology, radiological changes, and vascular endothelial growth factor (VEGF) immunostaining during bone healing. For this purpose, 24 New Zealand rabbits were assigned to three groups: the control group, the FM group, and the ketoprofen group. Our results revealed that there were no intraoperative complications, and all surviving rabbits achieved full-weight bearing. Significant periosteal reaction and callus formation were confirmed at 2 postoperative weeks. Interestingly, FM enhanced callus formation, bone union, and remodeling in the FM group compared to the control and ketoprofen groups. FM enhanced bone healing through early collagen deposition and marked angiogenesis process activation by increasing the expression of VEGF. Our findings demonstrated, for the first time, the potential imperative action of FM in the bone healing process rather than other NSAIDs in animals.

## 1. Introduction

The bone healing process involves multi-sequential events associated with numerous cellular functions that promote mineralization of the fracture site followed by remodeling to return the affected bone to its original structure [1]. In the literature, many postoperative drugs, including antibiotics [2] and anti-inflammatory drugs [3,4,5,6], have been used in different stages of the healing process. Among them, anti-inflammatory drugs have been extensively used as a painkiller to ensure fracture site immobility. However, numerous studies have demonstrated that steroidal and nonsteroidal anti-inflammatory drugs (NSAIDs) inhibit healing in soft and hard tissues [3,4]. Furthermore, various reports have suggested that NSAIDs interfere with bone healing, though others have contradicted these findings [7]. Celecoxib [8,9] and aspirin [10] inhibit bone healing [11,12], while ibuprofen [13] and parecoxib [14] do not affect bone healing.

The inhibitory action of NSAIDs might result from the inhibition of prostaglandin production from arachidonic acid via either the cyclooxygenase 1 (COX-1) or cyclooxygenase 2 (COX-2) enzyme pathway [15,16]. COX-1 is initially involved in physiological functions such as the preservation of hemostasis and gastric protection [17]. Meanwhile, COX-2 is initially found and involved in the pathophysiological pathways such as inflammation, fever, and pain [3,18]. However, prostaglandins play an important role in the bone healing process, especially through the regulation of osteoblast and osteoclast functions [19]. The marked inhibition of prostaglandin production decreases bone formation rate [20].

Ketoprofen is a potent nonselective COX-1 inhibitor that is extensively used in small animal medicine [15]. Ketoprofen is a nonselective NSAID used to decrease inflammation, pain, and fever, and it has analgesic effects. Data regarding the effect of ketoprofen on bone healing have been variable and controversial. Ketoprofen may not affect bone healing, especially in small doses for a short time [21]. In a study of the effects of ketoprofen on lumbar spinal fusion healing, It was revealed that using a single dose after intertransverse spinal fusion has no effect on bone healing [22]. Flunixin meglumine (FM) is a nonselective NSAID used as a painkiller, antifever, anti-inflammatory, and antitoxemic [23,24,25]. Few studies have explored the effect of FM on the soft tissue healing process. A previous study reported that FM promotes tendon healing by increasing fibroblast and blood supply to the wound site [23]. To the best of our knowledge, there has been no study on the effect of FM on bone healing. Growth factors play pivotal roles in the bone healing process [26]. The preservation of bone blood flow is considered one of the main mechanisms that promotes bone healing [26]. Vascular endothelial growth factor (VEGF) expression is essential for neurovascular development, i.e., vasculogenesis or the de novo production of blood vessels from mesenchymal precursor cells [26,27,28]. Given this information, the authors of this study aimed to determine the potential effects of ketoprofen and FM on bone healing in a rabbit femoral fracture model. The authors assessed these effects through a series of methods, including routine X-ray, histopathology, collagen deposition, and VEGF immunohistochemistry.

## 2. Materials and Methods

### 2.1. Ethical Committee Approval

The Research Ethics Committee of the Faculty of Veterinary Medicine, Kafrelsheikh University, reviewed and approved the procedures and protocols involved in both the animal welfare and surgical techniques of this study under approval number KFS-2020/7. The ethical approval code number is KFS-2020/7.

### 2.2. Animals

A total of 24 New Zealand rabbits at ten weeks of age and with bodyweight ranging from 2000 to 2200 g were used in this study. Rabbits were individually housed in a battery cage system with a natural day/night cycle at 21–28 °C. Rabbits had free access to complete commercial rabbit diets (Ibex International Co., Ltd., Nubaria, Egypt) and clean water throughout the study period. Two weeks before the start of the study, the rabbits were acclimatized.

### 2.3. Surgical Procedures

**Figure 1:**
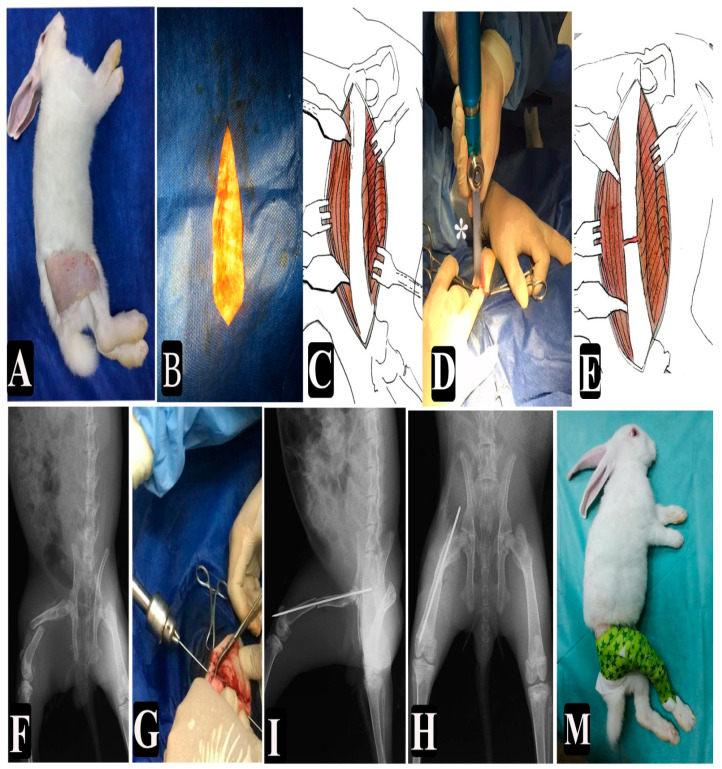
(**A**–**M**) Surgical procedures: (**A**) clipping and shaving of the femur region of the right limb of the rabbit, (**B**) draping and disinfection, (**C**) exposure of the femur bone, (**D**) making the fracture by the orthopedic saw ( * refers to fracture site in the midshaft of the femur), (**E**) a diagram showing the site of fracture in midshaft, (**F**) X-ray film directly after a fracture, (**G**) introduction of K-wire into the femur, (**I**) X-ray film lateral view, (**H**) X-ray film ventrodorsal view, and (**M**) bandage application.

Figure 1 illustrates the surgical procedures. Rabbits were anesthetized via the intramuscular injection of 5 mg/kg^−1^ bwt of xylazine hydrochloride (Xylaject, Adwia, New Cairo, Egypt) and 40 mg/kg^−1^ bwt of ketamine hydrochloride (Ketamine, Sigma, Cairo, Egypt) [23,29]. The right hind limb of each rabbit was prepared for aseptic surgical intervention. After a craniolateral skin incision, the biceps femoris was caudally retracted to expose the vastus lateralis muscle. The fascial septum of the vastus lateralis was incised, followed by a reflection of the vastus lateralis from the surface of the femur to expose the femoral diaphysis [30,31]. The fracture was made via the exposure of the femoral diaphysis using the surgical Saw Bojin BJ4101, and K-wire (size 1.2 mm) was introduced into the bone for fixation. The suturing of the muscle layer was performed with simple continuous absorbable polyglycolic acid sutures that were braided and coated with violet 4/0 (SURGICRYL-PGA, SMI AG, Vith., Belgium), and then the skin was sutured using simple interrupted absorbable polyglycolic acid sutures that were braided and coated with violet 2/0 (Surgicryl PGA, SMI AG, Vith., Belgium) [30]. The bandage was applied directly after surgery and kept for one week using a soft cotton pad, tongue depressor, gauze, and self-adhesive tape. All groups were injected with 10000 IU/kg bwt of penicillin and 10 mg/kg bwt of streptomycin (Pen & Strep Norbrook, West Sussex, UK) once daily Subcutaneous (S/C) for 5 successive days.

### 2.4. Animal Grouping

Animals were randomly divided into 3 groups as follows: a control group in which animals received saline solution as a placebo for 5 successive days, an FM group in which animals were intramuscularly injected with 2 mg/kg bwt of FMne (Finadyne®, MSD Animal Health, Walton, UK) once daily for 5 successive days, and a ketoprofen group in which animals were intramuscularly injected 2 with mg/kg bwt of ketoprofen (Ketolgin, Amoun, Al Obour, Egypt) once daily for 5 successive days [1,21,23].

### 2.5. Clinical Evaluation

Animals were clinically evaluated for body temperature, food intake, and daily activity during the week after surgery until external bandage removal. After this period, the animals were evaluated twice a week throughout the experimental period. The evaluation technique consisted of inspecting the external wound for dehiscence, edema, or pus; palpating the limbs by estimating the pain and muscular atrophy of the quadriceps muscle group of the operated limb; and comparing the operated limb with the contralateral limb [29].

### 2.6. Radiography

Radiography was performed using GXR 52 S, an X-ray apparatus (DRGEM Co., Ltd., Seoul, Korea). The X-ray beam was applied using the following factors: 40 KV, 25 MA, and 1.25 MAS. Two radiographic views (ventrodorsal and lateral) were taken. The X-ray was performed before and after surgical procedures at 0, 2, 4, 6, and 8 weeks after operation, as mentioned in [1].

### 2.7. Radiographic Scoring

The scoring system of this study was based on the point system described in [30]. It involved the quantitative assessment of periosteal reaction, bone union, and remodeling. Periosteal callus formation was given 4 when it was full, 3 for a moderate reaction, 2 for mild reaction, 1 for a minimum reaction, and 0 for no periosteal callus formation. The bone union was designated as 3 for complete bridging, 2 for moderate bridging more than 50% of the gap, 1 for slight union less than 50% of the gap, and 0 for nonunion. Bone remodeling was given 3 for advanced remodeling, 2 for moderate remodeling, 1 for early remodeling, and 0 for no remodeling. Images were scored by an independent radiologist who was blinded to the animal’s treatment.

### 2.8. Histopathology

Animals were euthanized by overdose anesthesia 4 and 8 weeks after the operation. At a rate of 4 specimens per each group (1 specimen/animal) during two time points of treatment, the specimens were subjected to the histopathological examination of osteogenesis at the fracture site using hematoxylin and eosin (H&E) stain and Masson’s trichrome stain [32]. Histopathological grading was performed according to the Lane and Sandhu histopathological scoring system, modified by Heiple et al. [33] and Bigham et al. [34], who used 20-point grading score to assess proximal union, distal union, cancellous bone, cortical bone, and marrow (4 points for each category).

### 2.9. Immunohistochemical Procedures

The immunohistochemical staining procedures followed that of Saber et al., 2019 [35]. Sections were dewaxed and immersed in a solution of a 0.05 M citrate buffer (pH 6.8) for antigen retrieval. These sections were treated with 0.3% H_2_O_2_ and protein block, and they were incubated with polyclonal rabbit anti-VEGF antibody (cat. no. PA5-16754, dilution 1/100, Thermo Fisher Scientific, Waltham, MA, USA). After being rinsed with phosphate-buffered saline, they were incubated with a goat anti-rabbit secondary antibody (cat. no. K4003, EnVision+™ System Horseradish Peroxidase Labelled Pomer, Dako, Thermo Fisher Scientific, Waltham, MA, USA) for 30 minutes at room temperature. Slides were then visualized with a DAB kit and eventually stained with Mayer’s hematoxylin as a counterstain. The staining intensity was assessed on a threshold basis using ImageJ Analysis software (NIH, Bethesda, MD, USA) and is presented as the percentage of positive area per mm^2^ in about 8 high power fields [35].

### 2.10. Statistical Analysis

Data were analyzed using GraphPad Prism 6 statistical software (GraphPrism Software, La Jolla, CA, USA). The statistical significance among groups was tested by a one-way analysis of variance (ANOVA) using the nonparametric Kruskal–Wallis H test followed by Dunn’s multiple comparison test. The *p* value was considered significant at less than 0.05.

## 3. Results

### 3.1. Radiographic Findings

**Figure 2:**
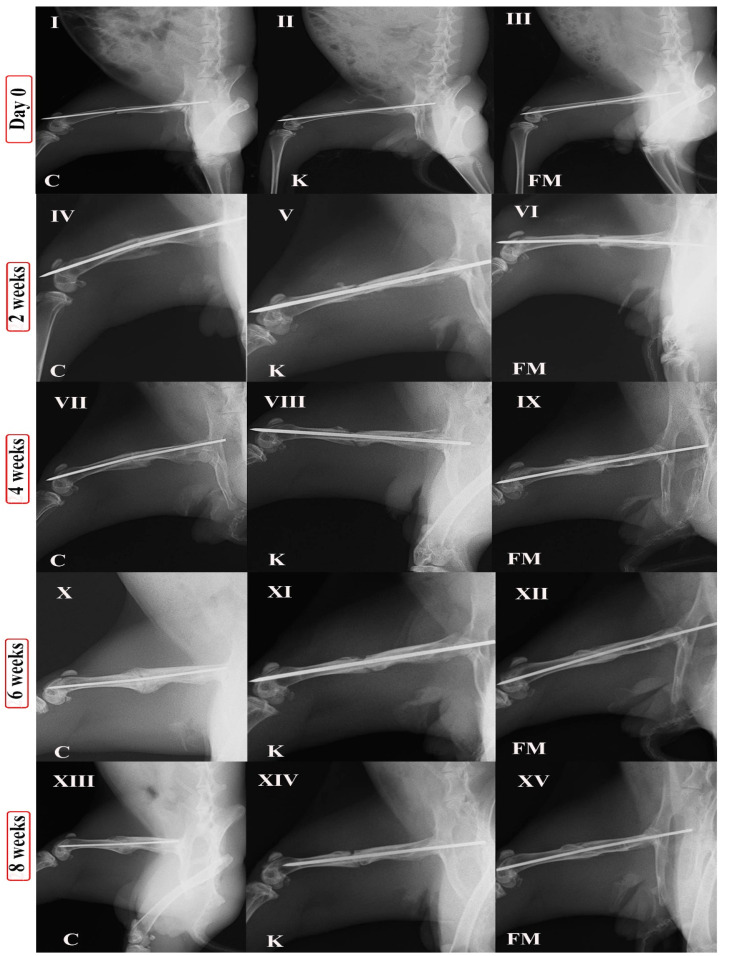
X-ray at different times (0, 2, 4, 6, and 8 postoperative weeks). C indicates the control group, K indicates the ketoprofen group, and FM indicates the flunixin meglumine group. (**I**,**IV**,**VII**,**X**,**XIII**) denote control group at different times 0, 2, 4, 6, and 8 weeks postoperatively. (**II**,**V**,**VIII**,**XI**,**XIV**) denote ketoprofen group at different times 0, 2, 4, 6, and 8 weeks postoperatively. (**III**,**VI**,**IX**,**XII**,**XV**) denote FM group at different times 0, 2, 4, 6, and 8 weeks postoperatively.

Table 1 presents the scores of radiographic findings according to the timeline. The radiographic findings of the control group at 2 postoperative weeks showed mild soft callus formation, with the noncomplete bridging bone union increasing over time. Meanwhile, the ketoprofen group revealed weak callus formation without a bone union. The FM group exhibited moderate soft callus formation with a noncomplete bridging bone union that increased over time (Figure 2). The callus formation score in the FM group (3.14 ± 0.51) was significantly higher (*p* < 0.05) than those in the ketoprofen group (2 ± 0.84) and the control group (2.5 ± 0.54). In stark contrast, callus formation was significantly lower (*p* < 0.05) in the ketoprofen group (2 ± 0.84) than that in the control group (2.5 ± 0.54). Importantly, the bone union score in the FM group (2.40 ± 0.40) was significantly higher (*p* < 0.05) than those in the ketoprofen group (0.83 ± 0.40) and the control group (1.66 ± 0.51). However, the bone union was significantly lower (*p* < 0.05) in the ketoprofen group (0.83 ± 0.40) than that in the control group (1.66 ± 0.51). At 4 postoperative weeks, the control group showed mild callus formation and bone union, with mild to moderate bridging between the bone edges. The ketoprofen group showed less bone callus formation than the other 2 groups and nonunion between the bone edges. The FM group showed greater mild soft callus formation than that in the control group and complete bone union. The callus formation score in the FM group (3.13 ± 0.51) was significantly higher (*p* < 0.05) than those in the ketoprofen group (1.66 ± 0.82) and the control group (2.67 ± 0.52). Callus formation was significantly lower (*p* < 0.05) in the ketoprofen group (1.66 ± 0.82) than in the control group (2.67 ± 0.52). The bone union score in the FM group (2.42 ± 0.41) was significantly higher (*p* < 0.05) than those in the ketoprofen group (1.17 ± 0.75) and control group (1.83 ± 0.75). In contrast, the bone union was significantly lower (*p* < 0.05) in the ketoprofen group (1.17 ± 0.75) than that in the control group (1.83 ± 0.75).

Based on the radiographic changes at 6 postoperative weeks, moderate callus formation was still present in the control group. The remodeling started in the few animals with moderate to complete bone union. Meanwhile, in the ketoprofen group, the minimal callus continued with a nonunion and no remodeling. However, the good callus started to decrease in size due to the early bone remodeling happening in approximately all FM rabbits with a complete bone union. The callus formation score in the FM group (2.29 ± 0.52) was significantly lower (*p* < 0.05) than that in the control group (3.17 ± 0.75). Moreover, the callus formation was significantly lower (*p* < 0.05) in the ketoprofen group (1.83 ± 0.75) than those in the control group (3.17 ± 0.75) and the FM group (2.29 ± 0.52). The bone union score in the FM group (2.46 ± 0.40) was significantly higher (*p* < 0.05) than those in the ketoprofen group (1.17 ± 0.75) and the control group (2.33 ± 0.52). Moreover, the bone union was significantly lower (*p* < 0.05) in the ketoprofen group (1.17 ± 0.75) compared to that in the control group (2.33 ± 0.52). Furthermore, the bone remodeling score in the FM group (1.33 ± 0.51) was significantly higher (*p* < 0.05) than those in the ketoprofen group (0 ± 0) and the control group (0.33 ± 0.52).

**Figure 3:**
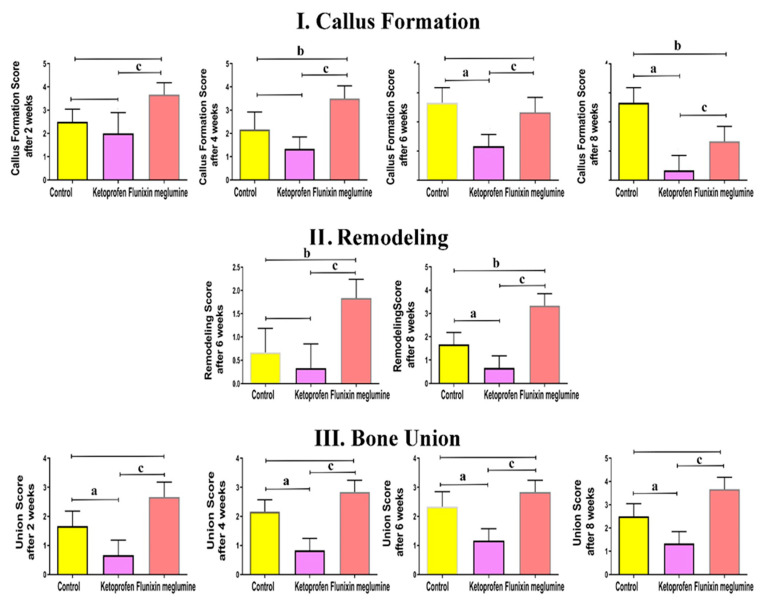
X-ray statistics at different times (2, 4, 6, and 8 postoperative weeks). (**I**): callus formation; (**II**): remodeling; (**III**): bone union. Data are expressed as mean ± SD. Superscript letters indicate the significance level: a denotes the significant difference between the ketoprofen and the control groups, b denotes the significant difference between the FM and control groups, and c: denotes the significant difference between the FM and the ketoprofen groups (*p* < 0.05).

The callus decreased in most animals in the control group due to remodeling, with a complete bone union at 8 postoperative weeks. However, the fractured bone was close to its normal shape in the FM group because of the great remodeling and good bone union. In the ketoprofen group, there was bad remodeling with nonunion. The callus formation score in the FM group (0.59 ± 0.52) was significantly lower (*p* < 0.05) than that in the control group (2.67 ± 0.52). Moreover, callus formation was significantly lower (*p* < 0.05) in the ketoprofen group (0.33 ± 0.52) than those in the control group (2.67 ± 0.52) and the FM group (0.59 ± 0.52). Additionally, the bone union score in the FM group (2.75 ± 0.41) was significantly higher (*p* < 0.05) than that in the ketoprofen group (0.33 ± 0.52) and the control group (2.66 ± 0.52). Moreover, the bone union was significantly lower (*p* < 0.05) in the ketoprofen group (0.33 ± 0.52) than that in the control group (2.66 ± 0.52). Likewise, the bone remodeling score in the FM group (2.33 ± 0.41) was significantly higher (*p* < 0.05) than those in the ketoprofen group (0 ± 0) and the control group (1.33 ± 0.52). Figure 3 depiects the X-ray statistics at different times (2, 4, 6, and 8 postoperative weeks). 

### 3.2. Histological Findings

#### 3.2.1. Fourth Week Study

**Figure 4:**
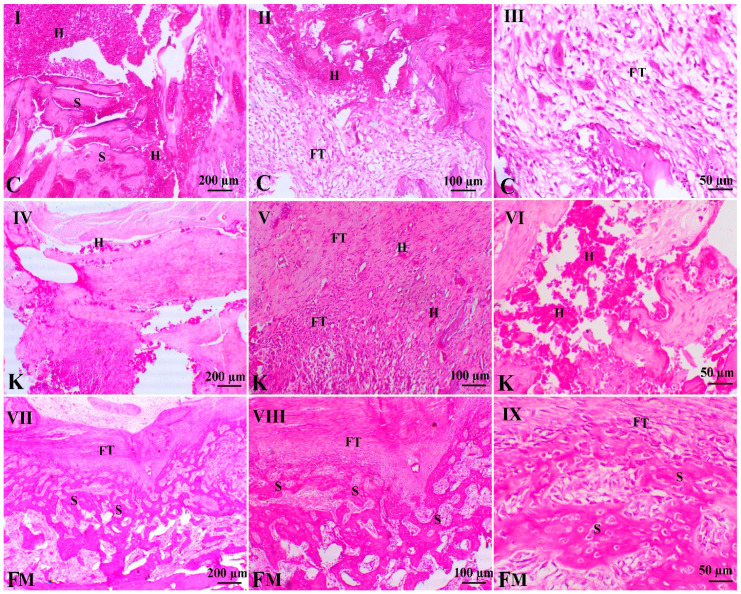
Photomicrographs of different H&E-stained femur sections of different groups (4 weeks). C indicates the control group, K indicates the ketoprofen group, and FM indicates the flunixin meglumine group. H indicates hemorrhage, FT indicates fibroblasts, and S indicates osseous tissue. Bar = 200, 100, and 50 µm. H&E, Bar = 200, 100 & 50 µm. (**I**,**II**,**III**) denote control group at different magnifications power. (**IV**,**V**,**VI**) denote ketoprofen group at different magnifications power. (**VII**,**VIII**,**IX**) denote FM group at different magnifications power.

As illustrated in Figure 4, control animals showed large hematoma with the still-defined junction of the two bone edges. The granulation tissues on both fracture sides showed entangled immature fibroblasts within an edematous matrix, with few collagens, and revealed the presence of few osteoblastic cells. Femur fracture in animals treated with ketoprofen demonstrated a marked decrease in inflammation, with the presence of cisterns of blood enclosed by fibrous connective tissues. Fractured animals treated with FM showed a marked decrease in inflammation signs, with a marked increase in the granulation tissue, revealing marked maturation of the fibrous connective tissues with a marked increase in osteoblastic activity. The newly osseous tissues were well-connected with the spongy bone.

#### 3.2.2. Eighth Week Study

**Figure 5:**
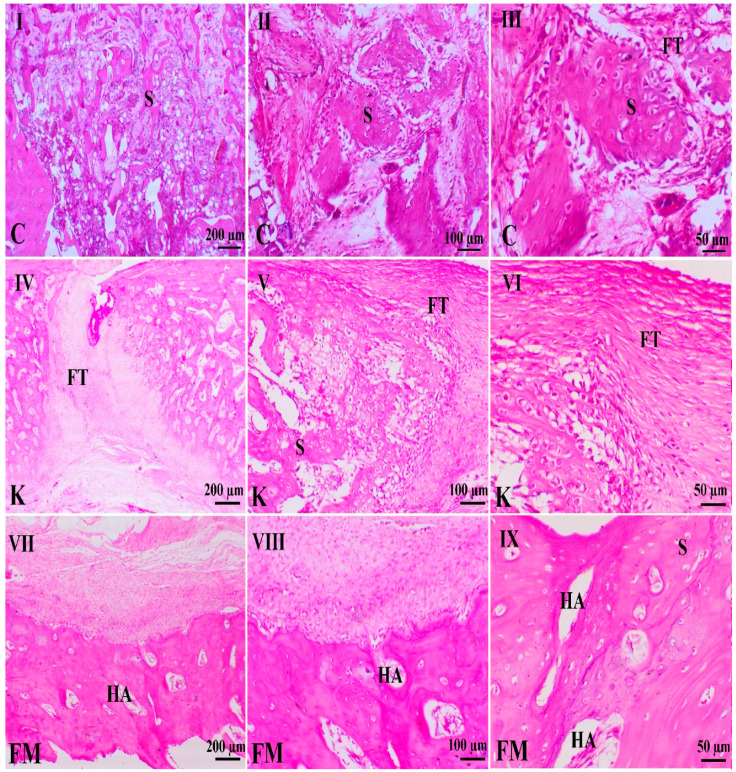
Photomicrographs of different H&E-stained femur sections of different groups (8 weeks). C indicates the control group, K indicates the ketoprofen group, and FM indicates the flunixin meglumine group. FM treated animals showed a marked bone fracture healing activity, while the ketoprofen group revealed fibrous band between both ends of fracture site without osteogenesis features in comparison with the control group (H&E stain). HA indicates haversian system, FT indicates fibroblasts and S indicates osseous tissue.. H&E, Bar = 200, 100 & 50 µm. (**I**,**II**,**III**) denote control group at different magnifications power. (**IV**,**V**,**VI**) denote ketoprofen group at different magnifications power. (**VII**,**VIII**,**IX**) denote FM group at different magnifications power.

**Figure 6:**
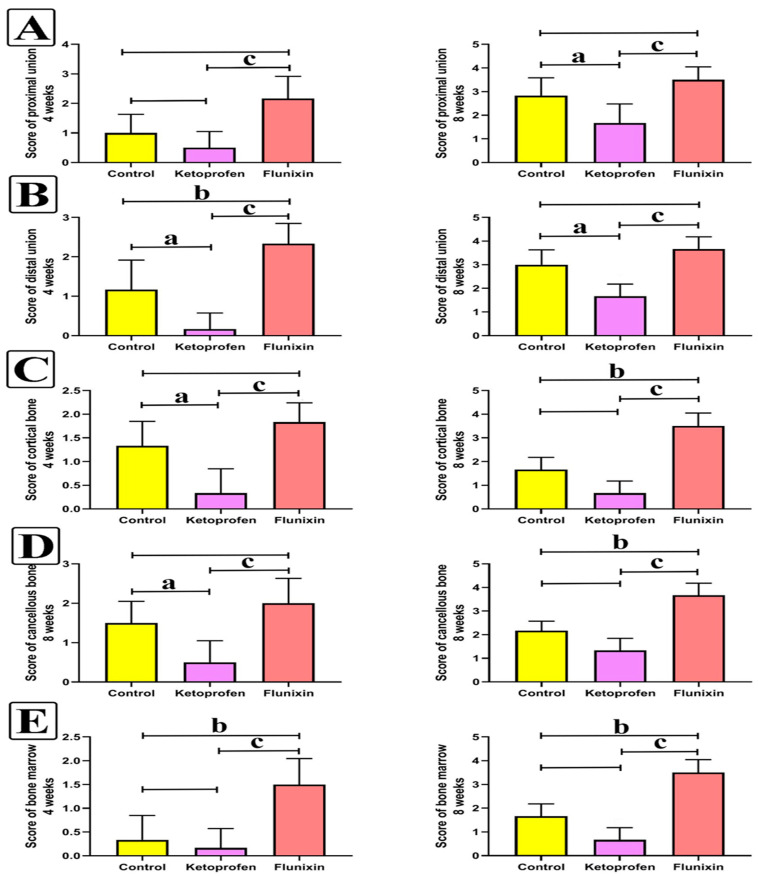
Histopathology statistics of H&E staining. (**A**): Score of the proximal union at 4 and 8 weeks. (**B**): Score of the distal union at 4 and 8 weeks. (**C**): Score of the cortical bone at 4 and 8 weeks. (**D**): Score of the cancellous bone at 4 and 8 weeks. (**E**): Score of the bone marrow at 4 and 8 weeks. Data are expressed as mean ± SD. Superscript letters indicate the significance level: a denotes the significant difference between the ketoprofen group and the control group, b denotes the significant difference between the FM group and the control group, and c: denotes the significant difference between the FM and the ketoprofen groups (*p* < 0.05).

As shown in Figure 5, animals in different groups showed various healing attempts. Control animals showed a decrease in hematomas and inflammation, with increased remodeling and osteogenesis. The cortical bones of both sides are related to immature osseous and cartilaginous callus within a fibro–hyaline matrix. The ketoprofen-treated animals showed a marked decrease in inflammation, with the presence of a gap filled with soft callus that consisted of granulation tissues with a small amount of osteoblastic cell activity. The FM-treated animals revealed mature, organized, thick bands of spongy osseous forming well-connected cortical bones covered with a thick periosteal layer. Figure 6 illustrates the quantitative scoring of bone healing within the four and eight weeks of the study. Marked early union, callus formation, and remodeling occurred in fractured animals treated with FM.

### 3.3. Masson’s Trichrome Staining

**Figure 7:**
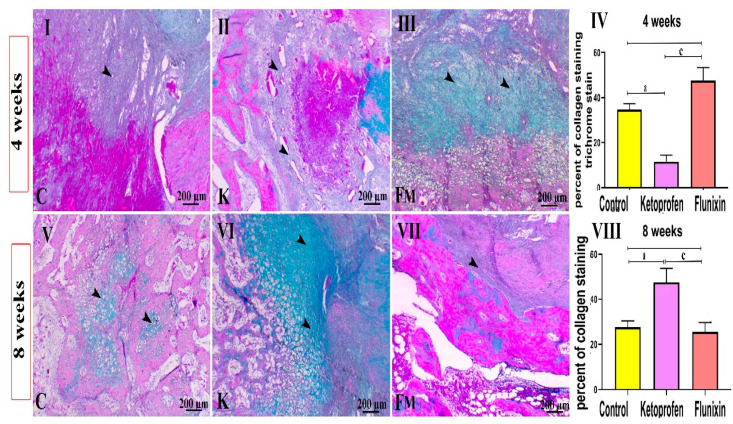
Photomicrographs of different Masson’s trichrome-stained femur sections of different groups (8 weeks). C indicates the control group, K indicates the ketoprofen group, and FM indicates the flunixin meglumine group. FM treated animals showed early fibroblastic activity, whereas the ketoprofen group still demonstrated pronounced fibrosis in the 8th week. Masson’s trichrome stain. Data are expressed as mean ± SD. Superscript letters indicate the significance level; a denotes the significant difference between the ketoprofen and the control groups; c: denotes the significant difference between the FM and the ketoprofen groups. (*p* < 0.05). (**I**,**V**) denote control group at 4, and 8 weeks. (**II**,**VI**) denote control group at 4, and 8 weeks. (**III**,**VII**) denote control group at 4, and 8 weeks. (**IV**) denotes statistical graph to the percent of trichrome staining at 4 weeks between control, ketoprofen, and FM groups. (**VIII**) denotes statistical graph to the percent of trichrome staining at 8 weeks between control, ketoprofen, and FM groups.

Results of Masson’s Trichrome Staining are shown in Figure 7. In the 4th week, the animals showed a variable amount of collagen. Control animals showed collagen deposition around the hematomas. Meanwhile, collagen deposition decreased on the margin of both sides of the fracture within the ketoprofen group. Interestingly, marked collagen deposition was visible within the FM-treated group (mostly within the early stage of bone healing), though it decreased in the late stage of bone healing. In the 8th week, the control and FM groups showed decreased collagen deposition with increased osseous tissues, while animals in the ketoprofen group showed late collagen deposition at the fracture sites. The quantitative scoring of collagen deposition of the 4th week’s study revealed a significant increase in collagen deposition within the FM group compared to the control group (*p* < 0.05). The ketoprofen group showed a significant decrease in collagen compared to the control group (*p* < 0.05). The 8th week’s study showed later collagen deposition in the ketoprofen group than that in the control and FM groups (*p* < 0.05).

### 3.4. Immunohistochemical Findings

#### VEGF Immunostaining

**Figure 8:**
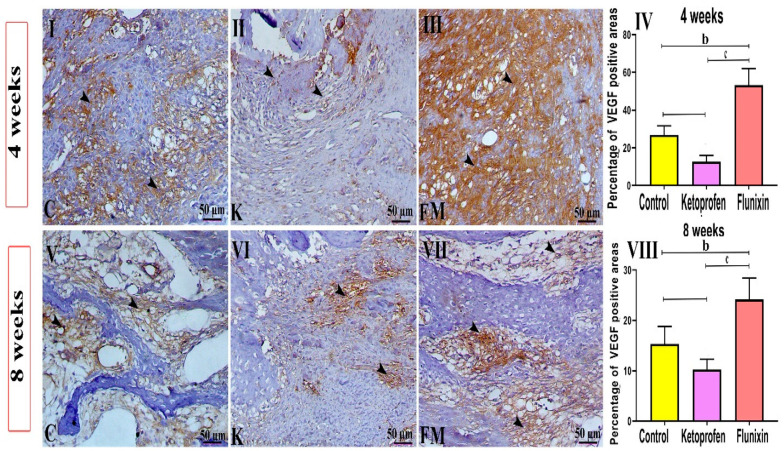
Photomicrographs of different VEGF IHC-stained tibial sections of different groups. C indicates the control group, K indicates the ketoprofen group, and FM indicates the flunixin meglumine group. Ketoprofen-treated animals showed a marked decrease in the expression of VEGF antibody, and FM-treated animals showed marked VEGF immunostaining (arrowheads indicate positive expression). Bar = 50 µm. Data are expressed as mean ± SD. Superscript letters indicate the significance level; b denotes the significant difference between the FM and control groups. c: denotes the significant difference between the FM and the ketoprofen groups. (*p* < 0.05) (**I**,**V**) denote control group at 4, and 8 weeks. (**II**,**VI**) denote control group at 4, and 8 weeks. (**III**,**VII**) denote control group at 4, and 8 weeks. (**IV**) denotes statistical graph to the percent of trichrome staining at 4 weeks between control, ketoprofen, and FM groups. (**VIII**) denotes statistical graph to the percent of trichrome staining at 8 weeks between control, ketoprofen, and FM groups.

As illustrated in Figure 8, the FM-treated group showed a marked expression of VEGF antibody within the soft callus tissue within the formed osseous cells and granulation tissues. Meanwhile, the ketoprofen-treated group showed a significant decrease in VEGF immunostaining within the granulation tissues in the 4th week study. In the 8th week, the expression was also increased within the bone marrow tissues in the FM group, whereas the ketoprofen group showed a mild expression of the VEGF within the hard callus tissues. The quantitative scoring of positive area expression of the VEGF antibody demonstrated a significant increase in VEGF immunostaining within the FM group at the 4th and 8th week studies compared to the control group (*p* < 0.05). There was also a significant decrease in VEGF expression within the soft and hard callus tissues of the ketoprofen group upon both sacrifice times (*p* < 0.05).

## 4. Discussion

Bone healing could be affected by multiple factors, such as postoperative treatment [36,37,38,39]. NSAIDs are commonly used postoperatively, but they might have diverse effects on bone healing [3,4,7]. Some delay the bone healing process [11,40,41], while others do not affect bone healing [12]. For the first time, this study has revealed the potential effect of one of the NSAIDs, FM, on bone fracture healing by enhancing angiogenesis at the fracture site, as reflected by VEGF expression and increasing osseous tissue formation. However, ketoprofen, like other NSAIDs, causes a delay in bone fracture.

Importantly, serial radiographs have been used to monitor the progress of bone fracture healing [42], including aspects of bone callus formation, bone union, and bone remodeling [30,43,44]. In this study, the radiographic findings revealed that FM improved fracture healing by improving callus formation, bone union, and bone remodeling. In stark contrast, ketoprofen delayed bone fracture healing by decreasing bone callus formation, bone union, and bone remodeling. The study’s findings are in harmony with previous studies that reported the delayed effect of ketoprofen on bone fracture healing in rabbits and cats [40,41].

Furthermore, good bone healing requires suitable stability, blood, and oxygen supply [44]. Several previous reports on different species (rabbits and rats) have shown the effect of FM on skin healing [24,45]. These studies proved that FM has adverse effects on the inflammatory phase of wound repair but not on the proliferative phase when fibroplasia is a major factor in wound strength [24]. Moreover, FM was not found to adversely influence breaking strength and wound contraction [45]. A previous study induced fibroblast proliferation in tendon injury, which fastened the tendon’s healing process [23]. In this study at 4 postoperative weeks, the histological results showed that FM had the same effect as that of the control and ketoprofen groups on osteoblast by increasing osseous tissues’ expression at the fracture site.

A bone matrix consists of nonorganic and organic components [46,47,48,49]. Collagen is the most copious protein in bone and represents approximately 90% of its organic matrix [49,50]. The collagen expression at a fracture site indicates osteogenesis of the mesenchymal stem cells (MSCs) [48,49]. In this study, the FM group showed a higher expression of collagen fibers at 4 weeks than those of the ketoprofen and the control groups, which were decreased by the effect of remodeling at 8 weeks.

In this study, ketoprofen stimulated fibroblast aggregation at the fracture site. The mechanism by which ketoprofen causes this fibroblast aggregation is still unknown and needs further research. It seems that it could be beneficial to soft tissue. However, in bone, it might hinder healing, as evidenced by the higher expression of fibroblast at the fracture site in the ketoprofen-treated group compared to the control and FM groups at the same stage (4 postoperative weeks). However, the study results contrasted with some previous reports that revealed that ketoprofen induced apoptosis in fibroblasts [51]. Furthermore, FM was found to enhance the healing process in contrast to other NSAIDs such as ketoprofen, which delayed this process through its influence on COX-1 and COX-2 enzymes. Some previous reports have revealed that prostaglandin E2 (PGE2) might regulate osteoblast behavior via the relative expression of the receptor activator of nuclear factor kappa-B ligand and osteoprotegerin, which is regulated through COX-1 and COX-2 enzymes. The inhibition of COX isozymes and the consequent decrease in PGE2 may be the mechanisms by which NSAIDs delays bone healing [4].

The available literature suggests that FM does not affect neovascularization, while ketoprofen reduces neovascularization [39,52,53]. More importantly, vascular supply at the fracture site is an essential step in the healing process since a good blood supply means plenty of the oxygen and nutrients required for fast healing [54]. Furthermore, the key physiological regulator of angiogenesis during embryogenesis and skeletal growth is VEGF [54,55]. A previous study revealed that FM stimulates angiogenesis in the wound site, enhancing tendon healing [23]. Similarly, in this study, FM was found to stimulate angiogenesis at the fracture site, which was obvious from the expression of VEGF at the fracture site in the FM group. However, no expression was reported in the other groups, suggesting that the FM group enhanced bone healing and the control and ketoprofen groups did not.

## 5. Conclusions

Given the present findings and their direct clinical applications, the use of FM after orthopedic surgery as a pain killer and anti-inflammatory is recommended. This study has proven for the first time that FM can enhance the bone healing process, callus formation, bone union, and remodeling in rabbits. Meanwhile, ketoprofen has an adverse influence on the bone healing process. The main limitations of the present study included its experimental fracture model that created osteotomy, significant violation of natural biology to cause the fracture, limited number of samples, short-term doses of ketoprofen, and lack of information regarding the detailed mechanisms by which ketoprofen delays healing. It would be interesting to investigate the effect of the short-term and long-term administration of ketoprofen on fracture healing to explain the main mechanisms by which ketoprofen may influence non-bone union and delay bone fracture healing. Future research to explore the mechanistic pathways involved in the underlying effects of FM on bone healing is also warranted.

## Figures and Tables

**Table 1:** Score of radiographic findings.

Timeline	Callus Formation			Bone Union			Bone Remodeling			*p* Value
	Control	Ketoprofen	FM	Control	Ketoprofen	FM	Control	Ketoprofen	FM	
2 weeks	2.5 ± 0.54	2 ± 0.84	3.14 ± 0.51	1.66 ± 0.51	0.83 ± 0.40	2.40 ± 0.40	-	-	-	*p* < 0.05
4 weeks	2.67 ± 0.52	1.66 ± 0.82	3.13 ± 0.51	1.83 ± 0.75	1.17 ± 0.75	2.42 ± 0.41	-	-	-	*p* < 0.05
6 weeks	3.17 ± 0.75	1.83 ± 0.75	2.29 ± 0.52	2.33 ± 0.52	1.17 ± 0.75	2.46 ± 0.40	0.33 ± 0.52	0 ± 0	1.33 ± 0.51	*p* < 0.05
8 weeks	2.67 ± 0.52	0.33 ± 0.52	0.59 ± 0.52	2.66 ± 0.52	0.33 ± 0.52	2.75 ± 0.41	1.33 ± 0.52	0 ± 0	2.33 ± 0.41	*p* < 0.05

## Data Availability

The data that support the findings of this study are available on request from the corresponding authors.
